# An Artificial Nerve Capable of UV‐Perception, NIR–Vis Switchable Plasticity Modulation, and Motion State Monitoring

**DOI:** 10.1002/advs.202102036

**Published:** 2021-10-29

**Authors:** Yao Ni, Jiulong Feng, Jiaqi Liu, Hang Yu, Huanhuan Wei, Yi Du, Lu Liu, Lin Sun, Jianlin Zhou, Wentao Xu

**Affiliations:** ^1^ Institute of Photoelectronic Thin Film Devices and Technology of Nankai University Tianjin 300350 P. R. China; ^2^ Key Laboratory of Optoelectronic Thin Film Devices and Technology of Tianjin Tianjin 300350 P. R. China; ^3^ Engineering Research Center of Thin Film Optoelectronics Technology of Ministry of Education Nankai University Tianjin 300350 P. R. China; ^4^ College of Electronic Information and Optical Engineering of Nankai University National Institute for Advanced Materials Nankai University Tianjin 300350 P. R. China; ^5^ College of Microelectronics and Communication Engineering Chongqing University Chongqing 400044 P. R. China; ^6^ No. 24 Research Institute of China Electronics Technology Group Corporation Chongqing 400060 P. R. China

**Keywords:** artificial neural network, broadband light, flexible organic‐heterojunction neuromorphic transistors, motion state monitoring, multiplexed‐neurotransmission signals

## Abstract

The first flexible organic‐heterojunction neuromorphic transistor (OHNT) that senses broadband light, including near‐ultraviolet (NUV), visible (vis), and near‐infrared (NIR), and processes multiplexed‐neurotransmission signals is demonstrated. For UV perception, electrical energy consumption down to 536 aJ per synaptic event is demonstrated, at least one order of magnitude lower than current UV‐sensitive synaptic devices. For NIR‐ and vis‐perception, switchable plasticity by alternating light sources is yielded for recognition and memory. The device emulates multiplexed neurochemical transition of different neurotransmitters such as dopamine and noradrenaline to form short‐term and long‐term responses. These facilitate the first realization of human‐integrated motion state monitoring and processing using a synaptic hardware, which is then used for real‐time heart monitoring of human movement. Motion state analysis with the 96% accuracy is then achieved by artificial neural network. This work provides important support to future biomedical electronics and neural prostheses.

## Graphical abstract

The first flexible organic‐heterojunction neuromorphic transistor (OHNT) that senses broadband light, including near‐ultraviolet (NUV), visible (vis), and near‐infrared (NIR), and processes multiplexed‐neurotransmission signals is designed. This provides new path to artificial nervous system with enriched functionalities and potentially applicable to bioinspired and biointegrated electronics.

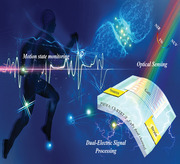

## 1 Introduction

Brainand sense organs acquire, transmit, and process information in a highly parallel manner.^[^ ^1^, ^2^, ^3^ ^]^ Neurons are connected by synapses, which communicate by exchange of neurotransmitters from the axon terminal across the synaptic cleft to the recipient dendrite; different types of neurons contribute to sensing, computing and learning,^[^ ^4^, ^5^, ^6^ ^]^ processing complex events at high efficiency and with low‐energy consumption. Thus, the integration of the multiple functionalities of sensing and processing into a simple neuromorphic device has become an important trend.

In many vertebrates, over 80% of information is detected by the visual sense.^[^ ^7^, ^8^ ^]^ The human retina possesses cone cells that detect light of specific wavelengths, and that preprocess information about the light's intensity before relaying the information to the brain. ^[^ ^9^ ^]^ Only a limited range of light is visible to humans; our retina cannot detect harmful ultraviolet (UV) or meaningful infrared (IR) signals. Thus, by emulating the retina, an artificial nerve that can detect and process broadband light stimuli would expand the human visual sense beyond visible light and be applicable to biomedical electronics and neural prostheses.

The motivation behind biologically inspired computing and learning architecture lies in the ability of such systems to continuously adapt to external stimuli that vary over time. ^[^ ^10^, ^11^ ^]^ Real‐time computing requires frequency‐sensitive short‐range plasticity, which can be reset in a very short time; learning that use cerebral neural networks is obtained by modulating the long‐range synaptic weights that are assigned to the connections between neurons, so the overall connectivity of the network can be reconfigured. To reproduce this ability of biological neurons to switch between short‐term and long‐term plasticity, artificial nerve systems require the construction of multiplexed neurotransmission of different neurotransmitters.^[^ ^12^, ^13^ ^]^ Depending on the nature of the stimulus, a neuron can produce different neurotransmitters that have different effects on the dendrite; this adaptability is the basis of an ability to accomplish complex functions and enables appropriate adaptation to changing conditions.^[^ ^14^ ^]^

Here, we present a flexible organic‐heterojunction neuromorphic transistor (OHNT) that emulates this adaptability. We constructed an organic p/n heterojunction by a low‐temperature processing method. This is the first report of an organic p/n heterojunction being developed for synaptic transistor, integrating broadband optical sensing and multiplexed electrical information processing. For near‐UV (NUV) perception, the device acts as a receptor with an ultralow power electrical energy consumption of several hundred attojoules per synaptic event; by altering near‐infrared (NIR) and visible (vis) light inputs, the device exhibits a switchable synaptic plasticity for recognition and memory. The heterojunction‐structured dual channel emulates co‐release of different neurotransmitters that induce short‐term plasticity (STP) or long‐term plasticity (LTP), analogous respectively to the responses to dopamine and noradrenaline in a biological neuron. The frequency‐dependent STP for the first time facilitates a human‐integrated real‐time monitoring of human movement on a synaptic hardware level, in the meantime solving the problem of weak‐tunable amplitudes in current mainstream heart monitoring equipment; the stable‐information‐storage LTP is used for pattern recognition of human photoplethysmogram (PPG) signals in body area sensor network (bodyNET) at 96% accuracy by artificial neural network (ANN). This provides a new path to artificial nervous system with enriched functionalities, and potentially applicable to bioinspired and biointegrated electronics.

## 2 Results and Discussion

**Figure 1:**
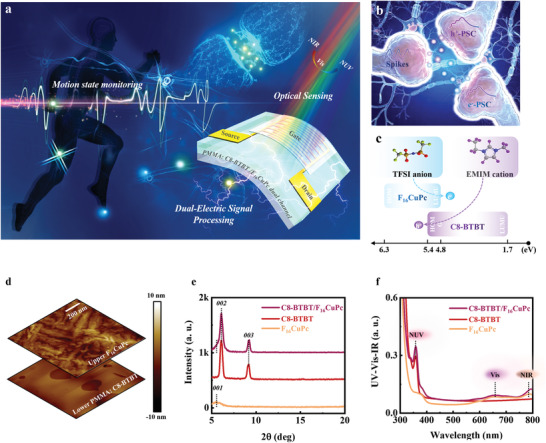
Schematic illustration and material characteristics. a) Schematic of an afferent nervous system that integrates optical sensing and dual‐electric signal processing units under electro‐optical pulse. b) Schematic of the transmissions of two kinds of excitatory neurotransmitters in the synaptic cleft under presynaptic spikes. c) Chemical structures of TFSI anion and EMIM cation in the ion gel and band diagrams of the dual channel; PSCs consisting of electrons (e^−^‐PSC) or holes (h^+^‐PSC) are generated in the F_16_CuPc or C8‐BTBT channel in response to two kinds of opposite presynaptic spikes. d) Atomic force microscope (AFM) images of lower PMMA: C8‐BTBT and upper F_16_CuPc films. e) XRD patterns and f) UV–vis–NIR absorption spectrum of F_16_CuPc, C8‐BTBT, and C8‐BTBT/F_16_CuPc heterojunction.

The OHNT integrated optical sensing and dual‐electric signal processing units (**Figure** **1**a). The light‐sensitive module is composed of two metal contact pads, and a phase‐separated poly(methyl methacrylate) (PMMA): 2,7‐dioctyl[1]benzothieno[3,2‐b][1]benzothiophene (C8‐BTBT)/ copper hexadecafluorophthalocyanine (F_16_CuPc) dual‐channel on a flexible PEN substrate; it can sense NIR, vis, and NUV, and convert these optical signals to distinct electric signals. In combination with ion gel, the OHNT can also mimic the release of dopamine and acetylcholine in a biological synapse, which can be used to implement real‐time monitoring and processing of heart signals in complex environments.

This OHNT emulated the interaction between dopamine and acetylcholine by switching the bipolar characteristics between holes and electrons; dopamine corresponds to hole and acetylcholine corresponds to electron (Figure 1b). The ion gel as the top gate contains 1‐ethyl‐3‐methylimidazolium (EMIM) cations and trifluoromethylsulfonyl (TFSI) anions; F_16_CuPc and C8‐BTBT are n‐type and p‐type channels, respectively (Figure 1c).^[^ ^15^, ^16^ ^]^ Application of positive or negative spikes drove anions or cations into the dual‐channel, where they attracted additional holes or electrons respectively in C8‐BTBT or F_16_CuPc channel to form electron‐induced postsynaptic current (e^−^‐PSC) or hole‐induced PSC (h^+^‐PSC) (Figure S1, Supporting Information). The PMMA: C8‐BTBT/F16CuPc contact shows a weak heterojunction effect which indicates a very small quantity of charges can be induced and the raise of off‐state current *I*_off_ can be effectively suppressed (Figure S2, Supporting Information).

A series of vapor/heating annealing processes enabled separation of a C8‐BTBT upper film that had the typical Stranski–Krastanov growth mode from a 105 nm thick PMMA: C8‐BTBT mixed layer,^[^ ^15^ ^]^ an ultrathin (5 nm) continuous F_16_CuPc film has little effect on injection of anions from the ion gel into the C8‐BTBT p‐type channel, but forms an additional n‐type channel (Figure 1d; Figures S3 and S4, Supporting Information). X‐ray photoelectron spectroscopy (XPS) spectra showed sharp peaks of S_2p_ and C_1s_ from PMMA: C8‐BTBT film; but F_1s_ and Cu_2p_ signals that are weak due to the extreme thinness of the F_16_CuPc film (Figure S5, Supporting Information).

The X‐ray diffraction (XRD) pattern of PMMA: C8‐BTBT/F_16_CuPc heterojunction film showed all of characteristic peaks of the two semiconductors (a peak at ≈5.6° for F_16_CuPc; peaks at ≈6.1° and ≈9.2° for C8‐BTBT) (Figure 1e).^[^ ^15^, ^16^ ^]^

NUV–vis–NIR and photoluminescence (PL) spectroscopies of different films were investigated (Figure 1f; Figure S6, Supporting Information). The PMMA: C8‐BTBT/F_16_CuPc heterojunction film combines the photoelectric characteristics of C8‐BTBT and F_16_CuPc (380, 640 and 790 nm).^[^ ^17^, ^18^ ^]^

**Figure 2:**
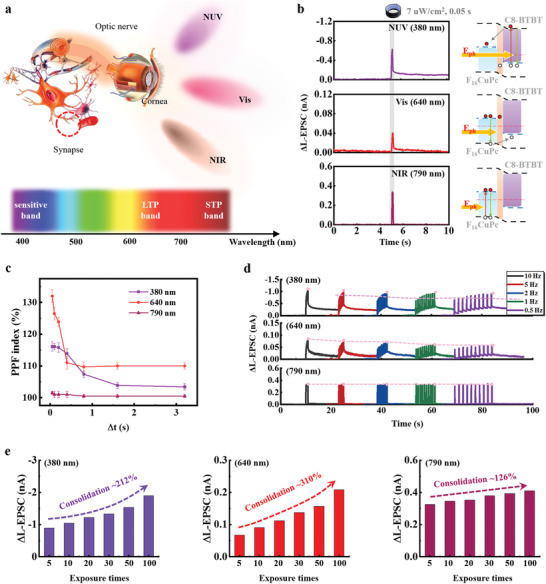
Broadband light perception and switchable synaptic plasticity. a) Artificial light‐perception afferent nerve composed of a sensing unit and a processing unit, which emulate the perception of lights at various wave lengths (NUV, vis, and NIR) and the conversion of photoelectric signals with high‐sensitive, LTP, and STP, respectively. b) (left) ∆L‐EPSCs (∆L‐EPSC = L‐EPSC − background current) for OHNT triggered by illumination at wavelengths of 380, 640, and 790 nm (*V*_DS_ for 380 nm = −1 V; *V*_DS_ for 640 and 790 nm = 0.2 V); (right) Energy band diagrams under three types of illuminations are on the right. c) PPF index according to the time interval, at different wavelengths of 380, 640, and 790 nm. d) ∆L‐EPSCs triggered by illuminations at different wavelengths of 380, 640, and 790 nm with different frequency. e) ∆L‐EPSCs triggered by different number of illuminations at different wavelengths of 380, 640, and 790 nm.

This heterojunction film is a light‐sensitive layer that is similar to the retina in a human eye (**Figure** **2**a). When used as an artificial afferent nerve for light perception, the OHNT can sense broadband light including NUV, vis, and NIR from the external environment and transmit signals to the synaptic cleft via the optical nerve. External rays of different wavelengths are integrated through synapses to achieve different characteristics of perception.

Under irradiation intensity of only 7 µW cm^−2^, the OHNT can respond to light of wavelengths 380 nm (NUV), 640 nm (vis), and 790 nm (NIR), and can generate light induced excitatory postsynaptic currents (L‐EPSCs) that have different strengths and types of plasticity (Figure 2b). The light pulses on p/n heterojunction channel were transformed into electrical signals. When a 790 nm light pulse of 0.05 s was applied, electron‐hole pairs in the F_16_CuPc channel layer were generated, and the current increased. However, it returned to its initial current level after around 0.1 s. This is because the photogenerated charge carriers, i.e., electrons and holes, as generated in the F_16_CuPc film easily recombined due to their symmetric distribution. Under the illumination of a 640 nm light pulse with a larger photon energy *E*_ph_, partial holes as generated in the F_16_CuPc layer can be injected into the C8BTBT channel region, so that some remaining electrons are possibly trapped in the F_16_CuPc channel and the C8BTBT/F_16_CuPc interface to cause an asymmetric charge carrier distribution, thereby reducing the charge carrier recombination rate to keep EPSC for an extended period.^[^ ^19^, ^20^ ^]^ Smaller peak value as compared with 790 nm light induced response may be due to weaker light absorption near 640 nm, consistent with UV–vis–NIR adsorption trends (Figure 1f). A 380 nm light pulse with a further increased *E*_ph_ can induce electron‐hole pairs in both the channels to result in even enhanced retention of EPSC.

Paired presynaptic illumination pulses with different pulse intervals Δ*t* yielded paired‐pulse facilitation (PPF) (Figure 2c).^[^ ^21^, ^22^ ^]^ . We calculated PPF index by using the EPSC peak as induced by the second spike (*A*_2_)/that induced the first spike (*A*_1_). The decay of the PPF index with the increase of Δ*t* fits the following double‐exponential equation with a rapid decay and a slowdecay^[^ ^23^ ^]^

(1) $$\mathrm{PPF}\;\mathrm{index}=C_{1}\;\times \exp\left(-\frac{\Delta t}{\tau_{1}}\right)+C_{2}\times \exp\left(-\frac{\Delta t}{\tau_{2}}\right)$$

where *C*_1_ (*C*_2_) and *τ*_1_ (*τ*_2_) are the initial facilitation magnitude and characteristic relaxation time of the rapid (slow) decay, respectively. As Δ*t* increased to 3.2 s, the PPF index under 640 nm was highest and remained stable value at ≈110%; but that under 790 nm quickly decreased to 100% within 0.1 s. This phenomenon indicates that the plasticity of vis‐triggered response is longer than that of NIR‐triggered response.

Application of a series of illumination spikes of different frequencies to OHNT can further confirm this conclusion (Figure 2d). As *f* of 380 nm and 640 nm illumination frequency increased, the current gain ΔL‐EPSC increased; however, under 790 nm illumination, variation in *f* caused no obvious change in ΔL‐EPSC.

We exposed the OHNT to different numbers of light pulses (0.05 s per exposure), then calculated the consolidation indexes (Figure 2e). After 100 pulses, the indexes were 212% under NUV, 310% under vis, and 126% under NIR. This artificial synapse shows a photoswitchable plasticity and in the meanwhile senses broadband light fully covering NUV, vis, and NIR (Table S1, Supporting Information).^[^ ^9^, ^23^, ^24^, ^25^, ^26^, ^27^, ^28^, ^29^, ^30^, ^31^, ^32^, ^33^ ^]^

**Figure 3:**
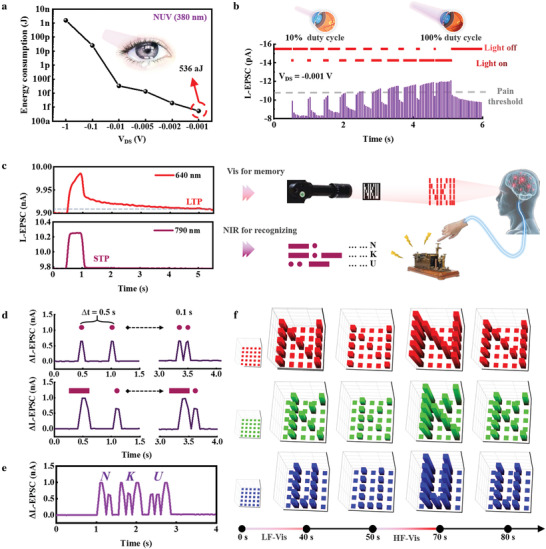
Light‐induced nerve functions: NUV‐receptor, IR‐induced recognition and vis‐induced memory. a) Electrical energy consumption according to the amplitude of *V*_DS_, at the wavelength of 380 nm. b) L‐EPSCs triggered by 380 nm UV light of different duty cycles, which simulates the proposed pain perception model caused by ultraviolet keratitis. c) LTP and STP triggered by 640 nm vis light, and 790 nm NIR light, respectively. d) ∆L‐EPSCs triggered by two pairs of spatiotemporally correlated 790 nm NIR light signals versus time. e) Optical wireless communication via OHNT with 790 nm NIR light signals representing the International Morse code of “NKU.” f) Presynaptic pulses programmed OHNT array with 640 nm vis light signals, emulating 5 × 5 pixel image of “NKU.”

Under NUV irradiation, response current decreased as the reading voltage decreased gradually. When the reading voltage was reduced to an exceptionally low level of only −0.001 V, the OHNT still showed a current gain of ≈2 pA (Figure S7, Supporting Information); the lowest electrical energy consumption of the OHNT was 536 aJ per synaptic event (**Figure** **3**a), which is much lower than by a biological synapse, and represents the lowest‐power UV‐optoelectronic neural device achieved to date (**Table** **1**).^[^ ^24^, ^26^, ^30^, ^34^, ^35^ ^]^

**Table 1:** Comparison of our work with previously reported opto‐electronic neuromorphic transistors in terms of the light intensity and electrical energy consumption under UV irradiation

Structure	Light wavelength	Light intensity	Electrical energy consumption	Year	Ref.
CsPbBr_3_ QDs	365 nm	0.153 mW cm^−2^	1.4 × 10^3^ pJ	2018	[22]
Si nanocrystals	375 nm	2.68 mW cm^−2^	0.14 nJ	2019	[32]
CsPbBr_3_ perovskite quantum dots	400 nm	100 µW cm^−2^	4.1 pJ	2019	[33]
Black phosphorus	365 nm	3 mW cm^−2^	9.24 × 10^2^ pJ	2019	[24]
NT‐CN /PMMA /pentacene	365 nm	0.38 mW cm^−2^	18.06 fJ	2020	[28]
C8‐BTBT /F_16_CuPc	380 nm	7 µW cm^−2^	536 aJ		This work

Detection of UV duty cycles and the resultant pain is important to avoid eye damage. Here, a pain perception model with a threshold (Corresponding to L‐EPSC > 11 pA) is proposed. At an ultralow reading voltage of −0.001 V, we used OHNTs to emulate receptors. A simulated cornea was exposed to NUV rays of different duty cycles (from 10% to 100%) to achieve different nociceptive characteristics (Figure 3b). L‐EPSC gradually increased as the duty cycle increased, and the pain threshold was easily surpassed; this result is similar to the process of physiological pain perception, in which an increase in harmful stimuli increases the degree of damage.

The current response of the OHNT under continuous illumination was compared at wavelengths of 640 and 790 nm (Figure S8, Supporting Information). As illumination duration increased from 0.05 to 0.5 s, 640 nm vis triggered an L‐EPSC that was stable and increased continuously, whereas 790 nm NIR triggered an L‐EPSC that stopped increasing at duration of 0.1 s. The switchable plasticity between LTP and STP by altering wavelengths yielded an ability to switch between functions, including memory and recognizing (Figure 3c, Figure S9, Supporting Information).

By converting 790 nm NIR light signals of International Morse code to distinct L‐EPSC signals, the OHNT provides an optical wireless communication method for human‐machine interfaces, in which every letter, number, and symbol can induce a distinct L‐EPSC amplitude response (Figure S10, Supporting Information). Due to the short‐term response as modulated by 790 nm NIR pulses, the OHNT device exhibited the high fault tolerance: commands were identified accurately even with ∆*t* = 0.1 s between the two light inputs (Figure 3d; Table S2, Supporting Information).^[^ ^3^, ^36^, ^37^, ^38^ ^]^ With ultrashort ∆*t* = 0.1 s, we input three letters “NKU” by using NIR, and received the complete information in only 2 s (Figure 3e).

This OHNT can also realize a transition from short‐term memory to long‐term memory under 640 nm vis. To exploit this memory characteristic of the device in vis illumination, we simulated a 5 × 5 OHNT array for optical pattern sensing and memory; the letters “NKU” were input to the array (Figure 3f). As light pulse rate increased the synaptic weights increased rapidly, and information could be efficiently retrieved for 10 s, with slight loss.

Multiplexed neurotransmission, i.e., corelease of different neurotransmitters from distinct microdomains of the same axon, has recently been found to underlie functions of the ventral tegmental area in the midbrain, such as decision making, flexible approach behaviors, learning and memory formation.^[^ ^39^, ^40^, ^41^, ^42^ ^]^ In a complex environment, a C8‐BTBT/F_16_CuPc dual‐channel can transfer holes or electrons to emulate co‐transmission of dopamine and noradrenaline under different stimulations.

**Figure 4:**
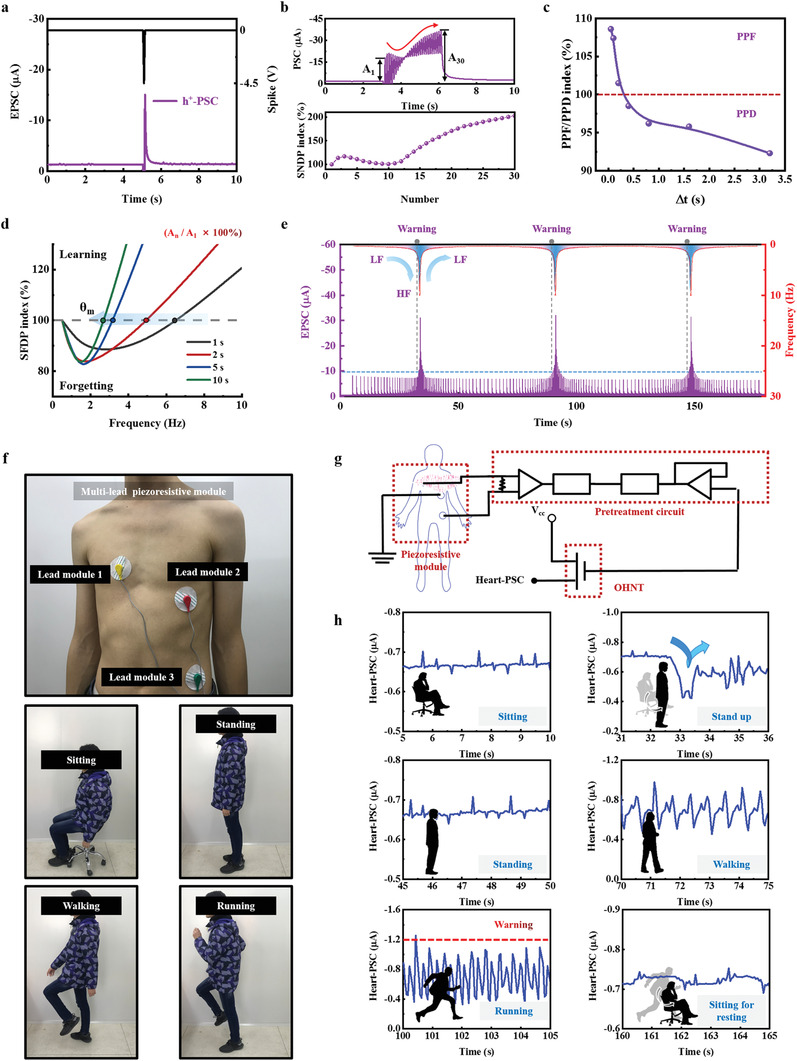
h^+^‐PSC based short‐term plasticity for a real‐time heart monitoring and alarming artificial nervous system. a) EPSC for OHNT triggered by negative spikes of −4.5 V (*V*_DS_ = −1 V). b) EPSC triggered by 30 consecutive negative spikes; SNDP index (*A*_n_/*A*_1_) according to the number of negative spikes from 1 to 30. c) PPF/PPD index according to the time interval. d) SFDP index as a function of the frequency of negative spikes after the OHNT has experienced different durations of spikes. e) 3 cycles of SFDP stimulated at the frequency of negative spike from 0.3 to 10 Hz. f,g) Schematic illustration of artificial nerve conduction system for **heart** signal processing, including piezoresistive module, pretreatment circuit and OHNT devices. h) **Heart**‐PSCs under different movements (sitting, stand up, standing, walking, running, sitting for resting).

When a negative spike of −4.5 V was applied to the ion gel, the h^+^‐PSC of OHNT was induced (**Figure** **4**a). As a series of repeated negative spikes was applied, the PSC showed a unique switch from inhibition to facilitation (Figure 4b). This nonmonotonic response to current may be due to delayed anion migration and ion‐to‐electron coupling. After different numbers of negative spikes, the currents returned to the baseline within < 10 s (Figure S11, Supporting Information). These results indicate that negative spikes can induce a stable and well‐defined STP.

PPF and paired‐pulse depression (PPD) are forms of STP in biological synapses. Application of paired presynaptic spikes yielded different paired PSC peaks (Figure S12, Supporting Information). The PPF/PPD index quantifies how Δ*t* between successive spikes affects the PSC change in the two‐phase behavior (Figure 4c). After two successive spikes were applied with Δ*t* < 0.3 s, the second PSC peak (*A*_2_) was higher than the first one (*A*_1_); this trend is analogous to PPF and could be explained as follows. The successive spikes drive a large number of anions into the channel in short time, and thus increased the efficiency of hole carrier transmission. At Δ*t* > 0.3 s, the index gradually changed to PPD, in which *A*_2_ was lower than *A*_1_; this change is caused by reduced influx of anions under infrequent action potentials. This response is analogous to the Bienenstock, Cooper, and Munro (BCM) response, in which the synapse can exhibit either potentiation or depression even when subjected to the same spike trains.^[^ ^43^, ^44^, ^45^ ^]^

In the BCM response, high‐frequency stimulation normally leads to potentiation and low‐frequency stimulation normally leads to depression, and at a threshold *f* = *θ*_m_ (*θ*_m_: SFDP index = 100%) the synaptic weight does not change. In our OHNT, as *f* increased from 1 to 10 Hz, the spike‐frequency‐dependent plasticity (SFDP) index first decreased, then increased (Figure 4d). For experience‐dependent synaptic plasticity (EDSP),^[^ ^46^ ^]^ both the amplitude and the sign of synaptic weight change depend on both the present stimulation conditions as well as the stimulation history. Here, after a period of increased synaptic activity (from 1 to 10 s), *θ*_m_ shifts to lower frequency, and thereby promotes synaptic potentiation such that spike trains that previously caused depression may now be above *θ*_m_ and cause potentiation instead.

Therefore, we applied series of negative spikes with *f* fluctuating back and forth between 0.3 and 10 Hz (Figure 4e). At *f* < 3 Hz, the h^+^‐PSC was relatively stable at <−10 µA; as *f* increased to > 3 Hz, the h^+^‐PSC increased exponentially, and the magnitude quickly exceeded −10 µA in a short time. This sensitive response characteristic of OHNT meets the needs of monitoring of heart rate during exercise: a heart rate should be constrained to be < 180 beats/minute; if it exceeds this speed, a warning will be triggered.

As a proof of physical utility, we constructed an artificial nerve‐conduction system that used a piezoresistive module, a pretreatment circuit and OHNT devices (Figure 4f,g). Heart rate signals collected by piezoresistive module were processed synchronously by pretreatment circuit, and then input to the OHNT as a real‐time stimulus. Different from current mainstream heart monitoring equipment that outputs a weak‐tunable amplitude of ≈1 mV,^[^ ^47^ ^]^ the device can reprocess the *f*‐dependent heart rate signals, then output amplitude‐dependent heart‐PSC for efficient real‐time monitoring.

A tester wore the artificial nerve conduction system and performed different movements for 200 s. The heart‐PSC of corresponding actions was collected at a sampling frequency of 20 Hz (Figure 4h; Figure S13 and Movies S1–S6, Supporting Information). With consideration of the influence of voltage bias in the cascade circuit, we reset the warning threshold of heart‐PSC as −1.2 µA. When the tester was sitting or standing, the heart‐PSC was far below threshold, although an obvious fluctuation occurred while performing the action of “stand up.” When the tester was walking, the amplitude of heart‐PSC increased, but it was still less than the threshold. When the tester was running, the amplitude of heart‐PSC vibration increased rapidly as the heart rate increased, and even temporarily exceeded the warning threshold at some instants; this response indicates that the tester may be at risk of overloading his heart. When the tester was sitting again after running, the heart‐PSC showed an envelope waveform that differed from the waveform that was observed while the tester was sitting for the first time; the difference occurs because the heart rate does not immediately return to normal after strenuous exercise.

**Figure 5:**
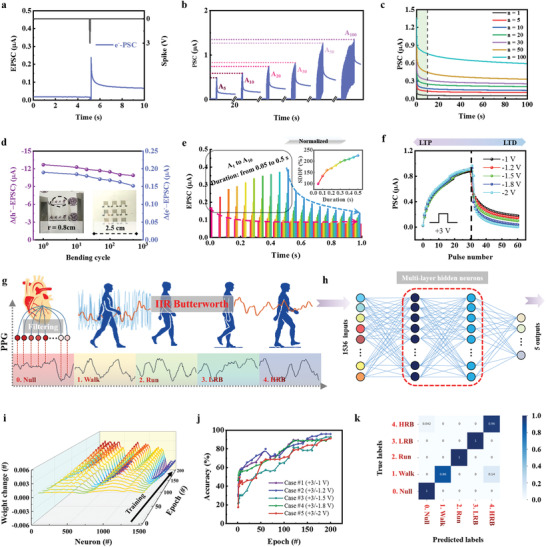
e^−^‐PSC based long‐term plasticity for exercise state recognition. a) EPSC for OHNT triggered by positive spikes of 3 V (V_DS_ = 0.2 V). b) EPSC triggered by different number of positive spikes. c) EPSCs with different number of positive spikes according to the degradation time. d) Trend of ∆EPSCs (∆EPSC = EPSC − background current) with bending cycles. The radius of curvature was 0.8 cm during the test. e) EPSCs triggered by positive spikes with different single‐spike durations; SDDP index (*A*_n_/*A*_1_) according to the duration of single spike from 0.05 to 0.5 s. f) Potentiation‐depression regulations. Synaptic potentiation triggered by a series of 30 positive spikes (4 V), and depression triggered by various series of 30 negative spikes with different amplitude (−1, −1.2, −1.5, −1.8, or −2 V). g) Neural network that uses OHNT array for PPG pattern recognition, in which 1536 input units and 4 output units are fully connected: state #1 = “walk,” state #2 = “Run,” state #3 = “Low‐resistance‐bike (LRB),” and state #4 = “High‐resistance‐bike (HRB)” to represent states of exertion. h) Diagram of a multilayer artificial neural network. i) Changes in the weight values of 1536 input synapses during 200 learning phases, under case #1. j) Convergence curves for the PPG pattern with respect to the number of learning epochs, under different cases. k) Confusion matrix for a classification test involving 125 PPG images after 200 epochs, under case #2.

To emulate the response to another neurotransmitter, the e^−^‐PSC was induced by applying a positive spike of 3 V for 50 ms to the ion gel (**Figure** **5**a). In this case, after a series of repeated stimuli, e^−^‐PSCs can be strongly increased, and persisted for > 100 s with stable states (Figure 5b,c); this process is different from h^+^‐PSC, and is analogous to the formation of LTP in biology.

We tested the stability of two types of PSCs in an OHNT array that had a side length of 2.5 cm, and contained nine identical dual‐plane‐gate transistors (Figure 5d). Before bending, a single device on this array had h^+^‐PSC of 12.7 µA and e^−^‐PSC of 0.19 µA. Two types of current peaks weakened after the OHNT was bent once to a radius of curvature of 0.8 cm, but retained > 80% of the initial current after 500 bending cycles.

Paired positive spikes were applied to obtain different paired e^−^‐PSC peaks (Figure S14, Supporting Information). As Δ*t* increased, the PPF index decreased gradually, but did not transform to PPD. This result indicates that due to the LTP, the second current peak will always be facilitated by the first one.

SDDP is considered to be an extended form of synaptic plasticity, and is involved in learning, associative memory, and forgetting.^[^ ^48^ ^]^ Repeated positive spikes with various duration from 0.05 to 0.5 s were applied to the OHNT (Figure 5e). The SDDP index (*A*_n_/*A*_1_) is introduced to demonstrate the trend that synaptic weights are generally enhanced with increased stimulation duration and the retention characteristic strengthened, because a long‐duration action potential increased cation migration and prevented back‐diffusion of cations in the ion gel/F_16_CuPc interface after the potential was removed.

Both potentiation and depression have been recognized as the biological basis at the cellular level for learning and memory activities.^[^ ^49^ ^]^ The OHNT devices were treated with 30 identical positive spikes, which elicited potentiation, then equal numbers of negative spikes, which elicited depression (Figure 5f). The PSC level with different linearity and symmetry could be controlled by adjusting the amplitude and polarity of input spikes. This ability can be used to conduct pattern recognition of human physiological signals in bodyNET.^[^ ^50^ ^]^

As a proof of concept, we selected a heartbeat dataset^[^ ^51^ ^]^ that is composed of five types PPGs categorized by different waveforms under different sports conditions. To interpret the PPG signals by using our OHNT array‐based neural network, we used the shape‐related features of each signal after it had been preprocessed with a sampling frequency of 256 Hz. We separated each PPG waveform into 1536 sequential electrical potentials, each with a duration of 6 s, then normalized each potential to a value between 0 and 255 inclusive. This network consists of 1536 inputs that correspond to the number of electrical potentials, and five output neurons that corresponding to the five PPG classes; the input and output layers are connected by multilayer hidden neurons with the backpropagation process. (Figure 5g,h; Figures S15 and S16, Supporting Information).

When each electrical potential in the PPG waveform is fed to each corresponding input in the neural network, the corresponding weight is repeatedly updated using the stochastic gradient ascent/descent learning algorithm (Figure 5i for case #1 during the learning epochs). We used 400 different PPG patterns for the inference test, under different cases. All accuracy rates exceeded 90% after 200 epochs (Figure 5j); for case #2, the accuracy was 96% (Figure 5k; Figure S17, Supporting Information), which yielded optimal weight update process in the neural network.

## 3 Conclusion

In summary, we fabricated a flexible artificial nerve that has an organic p/n heterojunction structure. This artificial nerve integrates optical sensing and electrical processing capabilities. The heterostructure expands the bandwidth of photosensitivity. To detect harmful NUV light, the OHNT acts as a pain‐perceptual receptor to sense different duty cycles of NUV light, with an ultralow electrical energy consumption of several hundred attojoules per event. By altering vis and NIR light inputs, switchable synaptic perception functions for recognition and memory were realized. For electrical information processing, the heterojunction‐structured dual‐channel enables selective charge‐carrier transport to emulate different neurotransmitters to cause STP or LTP. STP was useful for human‐integrated real‐time human heart signal processing to monitor human movements. LTP with stable storage states was used for pattern recognition of human PPG signals in bodyNET with 96% accuracy by ANN. A 3 × 3 array of the devices retained 80% of its initial current level after 500 cycles of bending at a radius of 0.8 cm. This work provides a new approach to enrich the function of artificial nervous systems and their application to cognitive neuromorphic electronics.

## Conflict of Interest

The authors declare no conflict of interest.

## Supporting information

Supporting Information

Supplemental Movie 1

Supplemental Movie 2

Supplemental Movie 3

Supplemental Movie 4

Supplemental Movie 5

Supplemental Movie 6

## Data Availability

The data that support the findings of this study are available in the Supporting Information of this article.
