# Supplementary material for: An Artificial Nerve Capable of UV‐Perception, NIR–Vis Switchable Plasticity Modulation, and Motion State Monitoring
Source: Adv Sci (Weinh). 2021 Oct 29;9(1):2102036. doi: 10.1002/advs.202102036 (PMC8728819; doi:10.1002/advs.202102036)
Supplement: Supplementary file 1 — Supporting Information [file ADVS-9-2102036-s003.pdf]

© 2021 Wiley-VCH GmbH

ADVANCED SCIENCE
Open Access

Supporting Information

for *Adv. Sci.,* DOI: 10.1002/advs.202102036

An artificial nerve capable of UV- perception, NIR-Vis switchable plasticity modulation, and motion state monitoring

*Yao Ni, Jiulong Feng, Jiaqi Liu, Hang Yu, Huanhuan Wei, Yi Du, Lu Liu, Lin Sun, Jianlin Zhou, and Wentao Xu**

Supporting Information

An artificial nerve capable of UV-nociception, NIR-Vis switchable plasticity modulation, and electrocardiogram information processing

*Yao Ni[1], Jiulong Feng[1], Jiaqi Liu[1], Hang Yu, Huanhuan Wei, Yi Du, Lu Liu, Lin Sun, Jianlin Zhou, Wentao Xu**

Y. Ni, J. Feng, J. Liu, H. Wei, Y. Du, L. Liu, Dr. L. Sun, Prof. W. Xu.
Institute of Photoelectronic Thin Film Devices and Technology of Nankai University, Tianjin 300350, P. R. China
Key Laboratory of Optoelectronic Thin Film Devices and Technology of Tianjin, Tianjin 300350, P. R. China
Engineering Research Center of Thin Film Optoelectronics Technology of Ministry of Education, Nankai University, Tianjin 300350, P. R. China
College of Electronic Information and Optical Engineering of Nankai University, National Institute for Advanced Materials, Nankai University, Tianjin 300350, P. R. China
Corresponding author. Email: wentao@nankai.edu.cn; bnuch@hotmail.com.

H. Yu
College of Microelectronics and Communication Engineering, Chongqing University, Chongqing 400044, P. R. China
No.24 Research Institute of China Electronics Technology Group Corporation, Chongqing 400060, China

Dr. J. Zhou
College of Microelectronics and Communication Engineering, Chongqing University, Chongqing 400044, P. R. China

Experimental Section

Fabrication of OHNT device. PMMA (0.4 wt%): C8-BTBT (2 wt%) mixed solution in chlorobenzene was spin-coated onto the PEN substrates at 4000 rpm/min for 40 s. The prepared sample was turned upside down and adhered to the open top of an upright beaker that held chlorobenzene, then the beaker temperature was increased to 120 °C and the chlorobenzene was allowed to evaporate for 1 h. PMMA spreading effect prevents C8-BTBT molecules from strong molecular interactions to aggregate into small dots, and directs the formation of plate-like organic C8-BTBT crystals. The sample was then annealed in nitrogen atmosphere at 60 °C. Then a 5-nm $F_{16}CuPc$ layer was deposited at a rate of 0.2 Å/s onto the PMMA: C8-BTBT layer in a vacuum under a pressure of 5×10^{-4} Pa. Subsequently, source and drain electrodes of the gold layer (60 nm) were prepared on the PMMA: C8-BTBT/$F_{16}CuPc$ film by thermal evaporation. Finally, the ion-gel top-gate dielectric (mass ratio between polymer Poly(vinylidenefluoride-co-hexafluoropropylene) [PVDF-HFP] and ionic liquid (1-ethyl-3-methylimidazolium bis(trifluoromethylsulfonyl)imide [EMIM-TFSI] was 1:3) layer was transferred onto the channel area. A tungsten (W) probe that contacted the ion gel was used to apply the pre-synaptic spike, which is the pre-synaptic input terminal.

Characterization and opto-electronical measurements. AFM images were obtained using a Bruker dimension icon microscope in tapping mode. Scanning electron microscope (SEM) images were performed using an FEI-Apreo field emission microscope. XPS was conducted using a Thermo Scientific (ESCALAB 250Xi). XRD patterns were obtained using a Rigaku Ultima IV instrument. The optical absorption spectra were performed using a UV-Vis-NIR spectrophotometer (Cary 5000) at room temperature. PL patterns were characterized with a fluorescence spectrometer (Edinburgh FS5) in ambient air. All electrical measurements were performed using a Keithley 4200A semiconductor parameter analyzer and a probe station in N_2 environment in a glove box at room temperature. The optical signal was generated using a Xenon source (Zolix, GLORIA-Bright) with 380-nm wavelength.

Testing the devices on human bodies. All human subjects involved in the heart signal tests provided informed consent, and the study protocol (no. NKUIRB2021044) was approved by the Institutional Review Board office at Nankai University. The skin shown in the figures and video are those of J. Liu, who has given his consent to publish these images and movies.

PPG simulations and statistical analysis: The simulator used a 4-layer (1536 × 256 × 64 × 5) perceptron neural network. The input layer with 1536 neurons, representing the PPG signals (1536 sampling points), was fully connected to dual-hidden layers of 256/64 neurons,

which was then fully connected to the output layer of 5 neurons. Softmax was used as the classification function of the output layer. For each synapse, all the weights were randomly in normal distribution (standard deviation = 0.1) and the gradient descent method and a mini-batch size of 125 were used to train the network. In the weight update method based on the hardware-based backpropagation algorithm, as the measured conductance states always have positive values, the synaptic weight is represented as the difference between the conductance values of two equivalent synaptic devices ($\Delta W = \Delta W_p - \Delta W_d$). To determine whether the synaptic weight was potentiated or depressed, we used a cross-entropy approach to calculated the loss between the output value (y_1) and each label value (d_t). The nonlinear behavior of the weight update is mapped to the piecewise weight decay, and the initial learning rate is 0.1.

Supplementary Figures

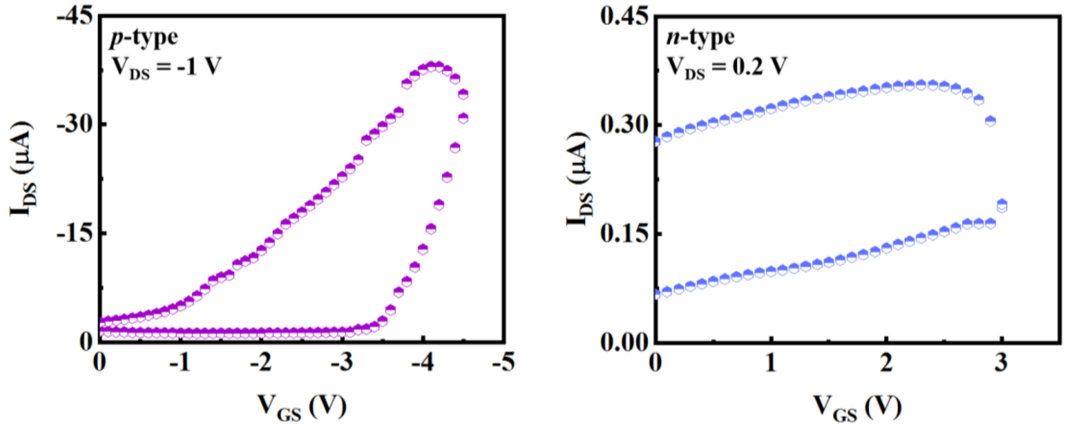

Figure S1. Transfer characteristics of a 3-terminal OHNT.

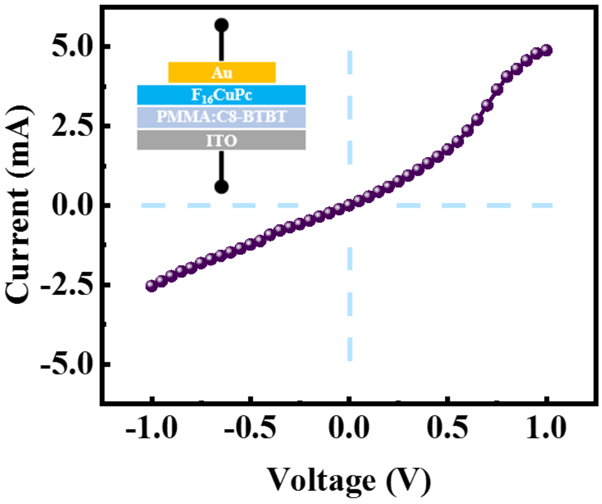

Figure S2. The rectification properties of the PMMA: C8-BTBT/$F_{16}CuPc$ junction.

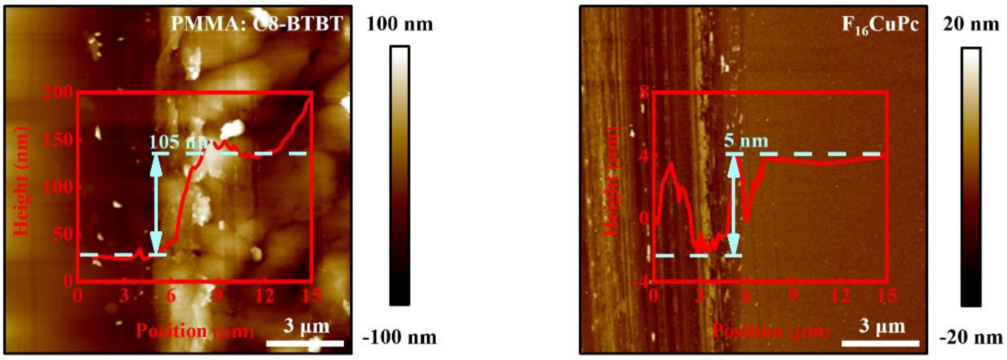

Figure S3. Atomic force microscopy (AFM) images with height profiles of PMMA: C8-BTBT and $F_{16}CuPc$ films.

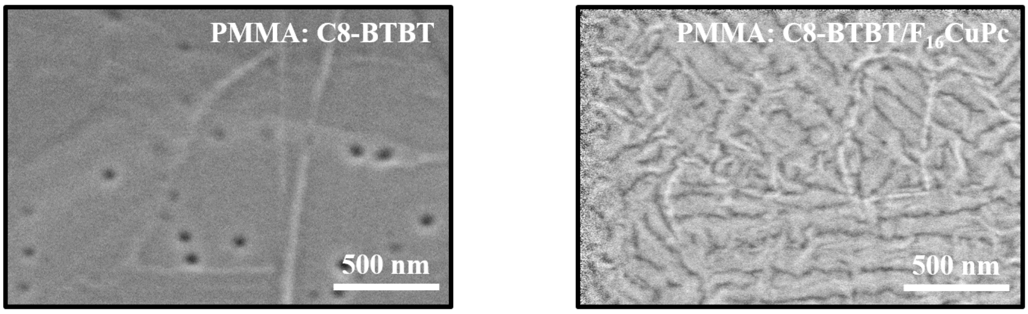

Figure S4. SEM images of PMMA: C8-BTBT and PMMA: C8-BTBT/F_{16}CuPc films on SiO_2 substrate.

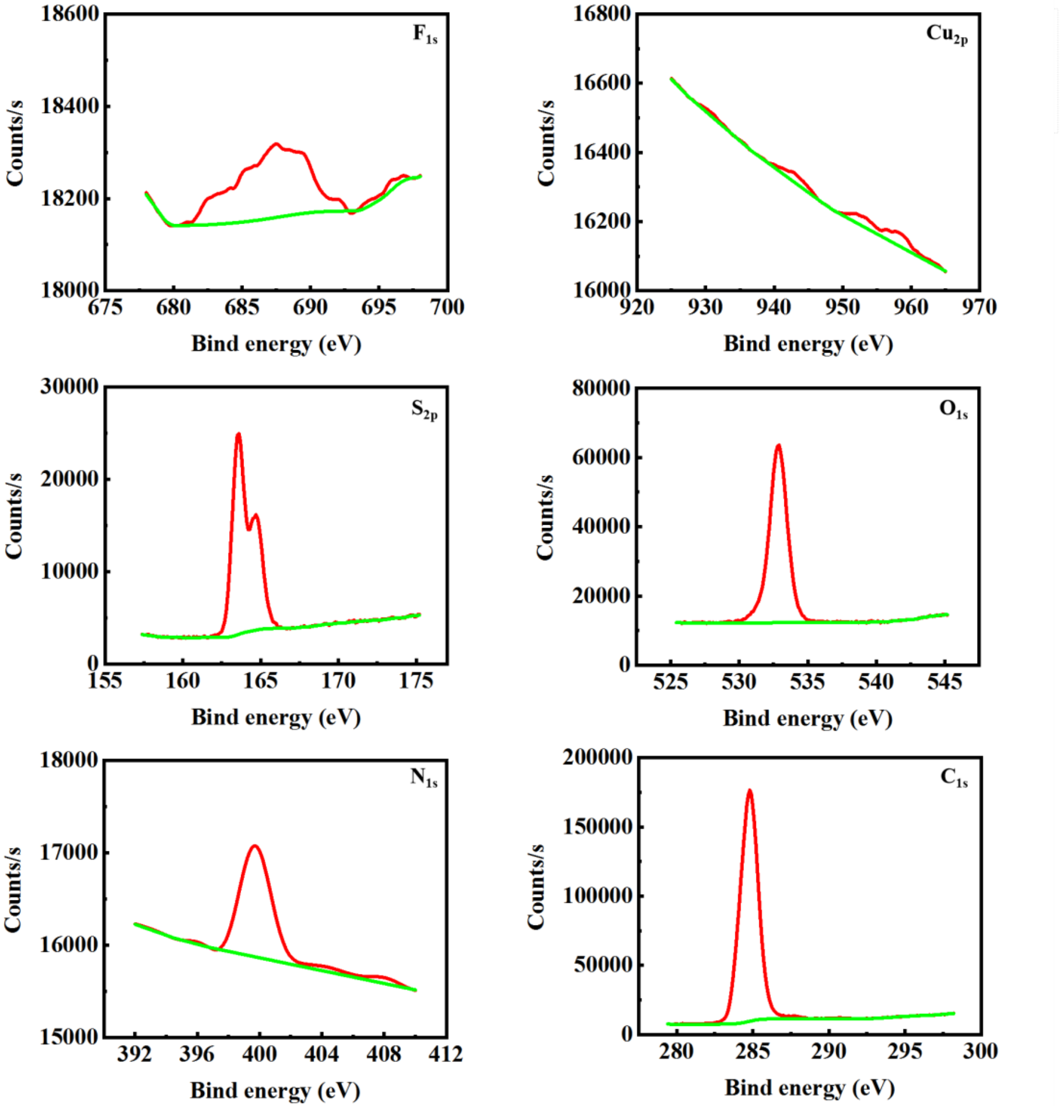

Figure S5. XPS survey spectra of F_{1s}, Cu_{2p}, S_{2p}, O_{1s}, N_{1s} and C_{1s} of the PMMA: C8-BTBT/F_{16}CuPc films.

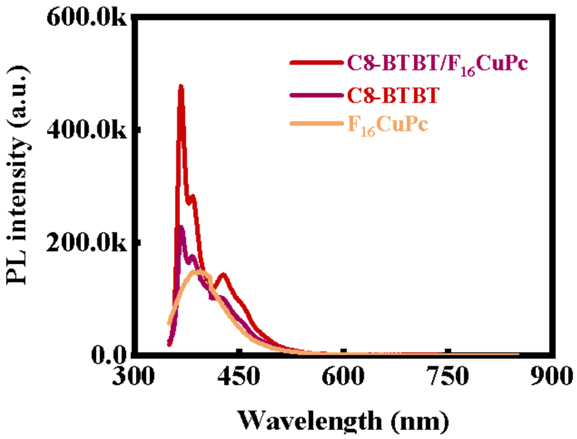

Figure S6. PL spectra of PMMA: C8-BTBT, $F_{16}CuPc$, and PMMA: C8-BTBT/$F_{16}CuPc$ films on glass substrate. PL excitation wavelength was 325 nm.

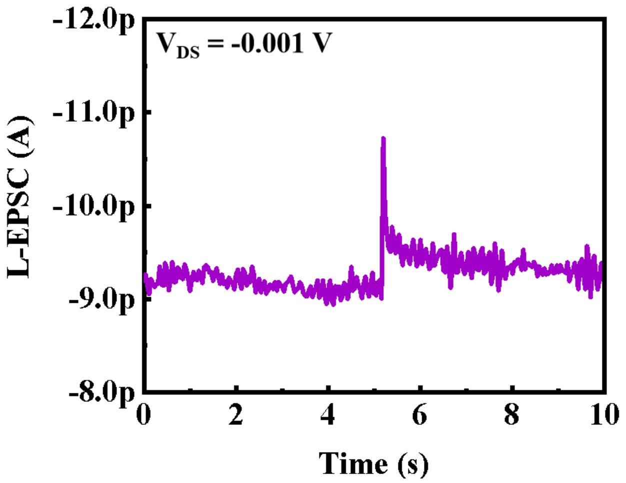

Figure S7. L-EPSC triggered by 380 nm NUV light, under V_{DS} = -0.001 V.

Note:

The electrical energy consumption for a single light pulse event is defined as $I_{peak} \times t \times V$, where I_{peak}, t, and V are the peak value of the current, the pulse duration time, and the pulse voltage, respectively.

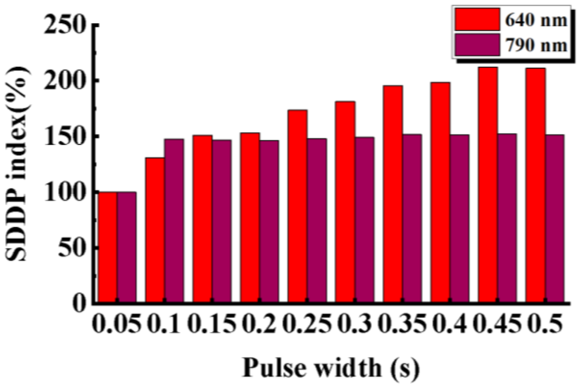

Figure S8. SDDP index according to the duration of single illumination from 0.05 to 0.5 s, at wavelengths of 640 and 790 nm.

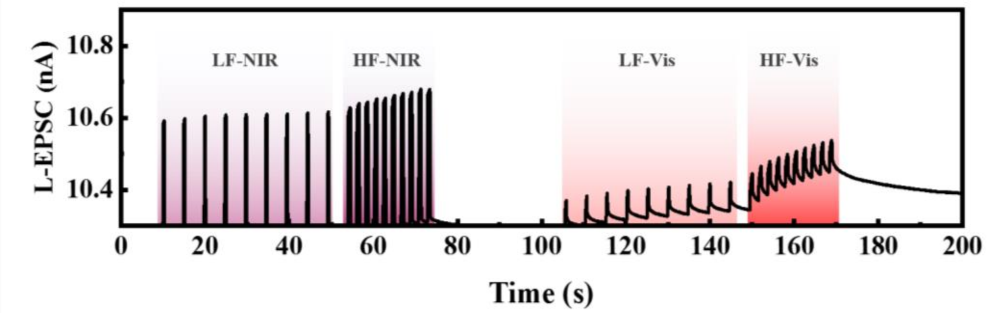

Figure S9. EPSC under low frequency (LF) and high frequency (HF) irradiation pulses of 640 nm Vis and 790 nm NIR lights, respectively.

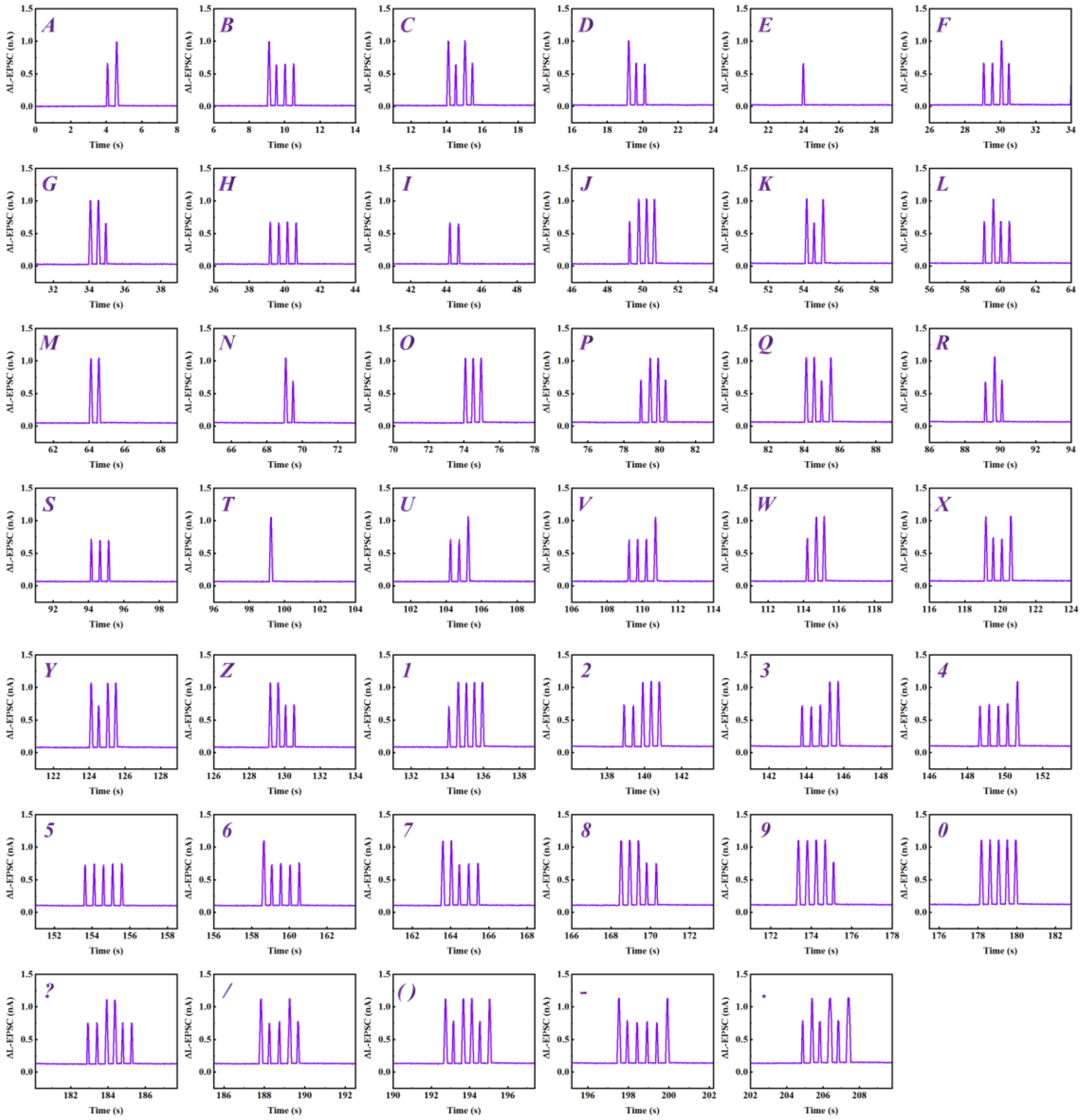

Figure S10. Optical wireless communication by OHNT with 790-nm NIR light signals representing the International Morse codes of 26 letters, 10 numbers, and 5 symbols.

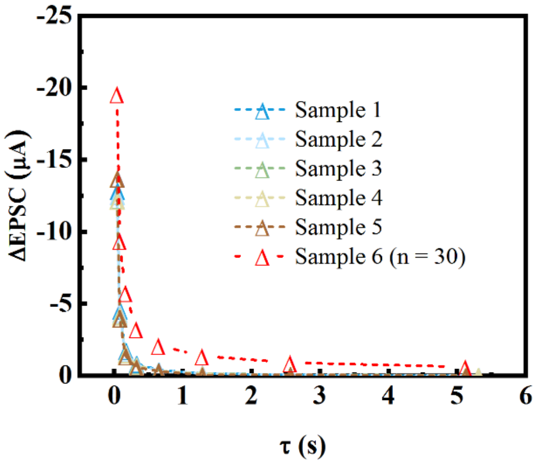

Figure S11. EPSCs (Samples 1-5 with single negative spike; Sample 6 with 30 negative spikes) according to the degradation time.

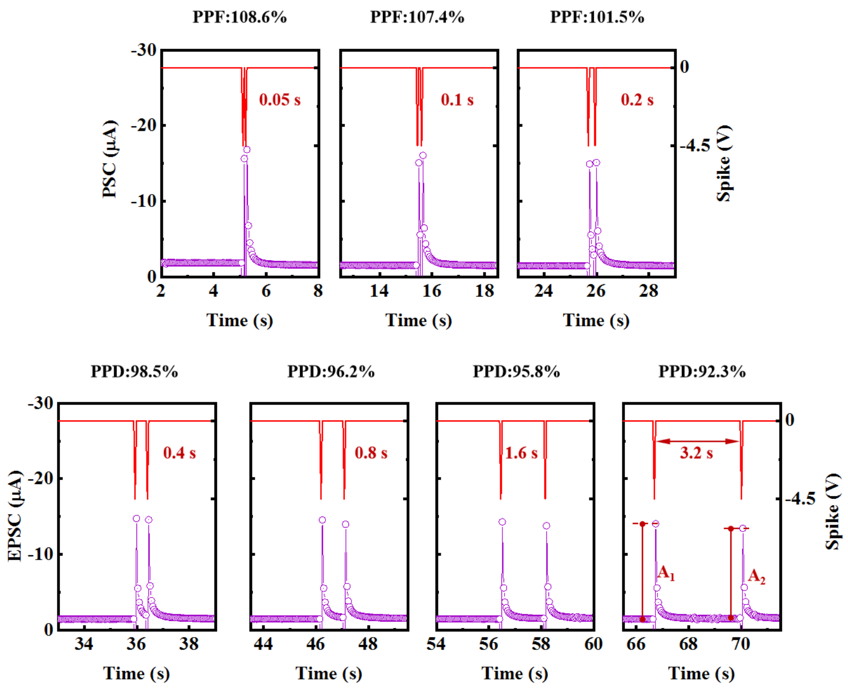

Figure S12. EPSCs triggered by two consecutive negative spikes separated by intervals from 0.05 to 3.2 s;

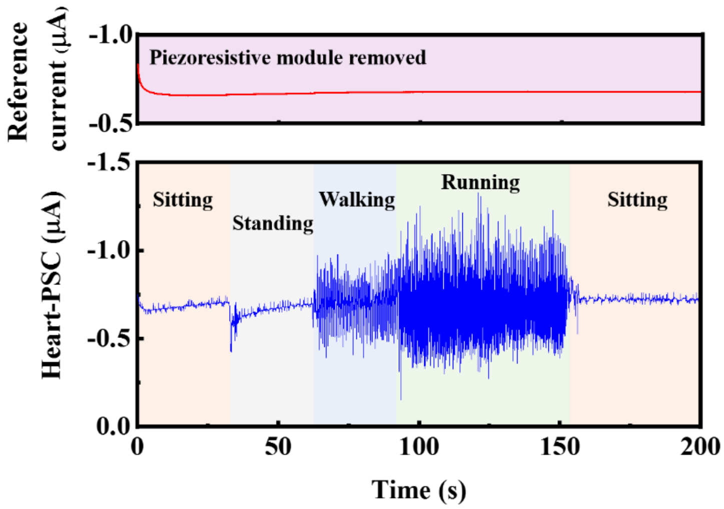

Figure S13. Detailed **heart**-PSCs under different movements.

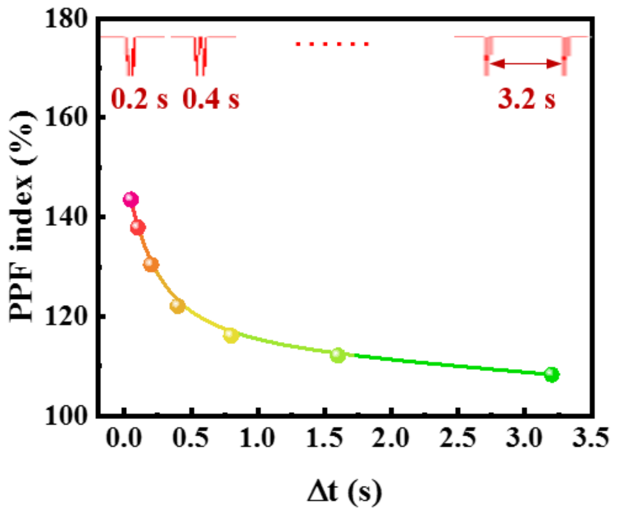

Figure S14. Positive spikes triggered PPF index by positive spikes according to the time interval.

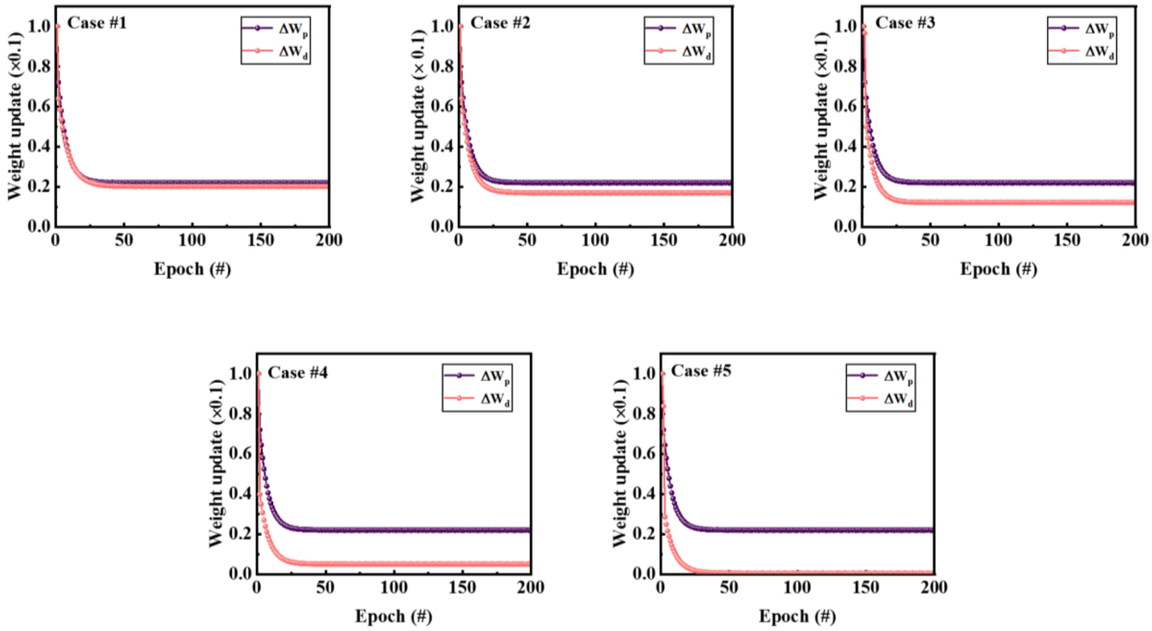

Figure S15. The fitting ΔW_p and ΔW_d with different cases (case #1: 4/-1 V; case #2: 4/-1.2 V; case #3: 4/-1.5 V; case #4: 4/-1.8 V; case #5: 4/-2 V).

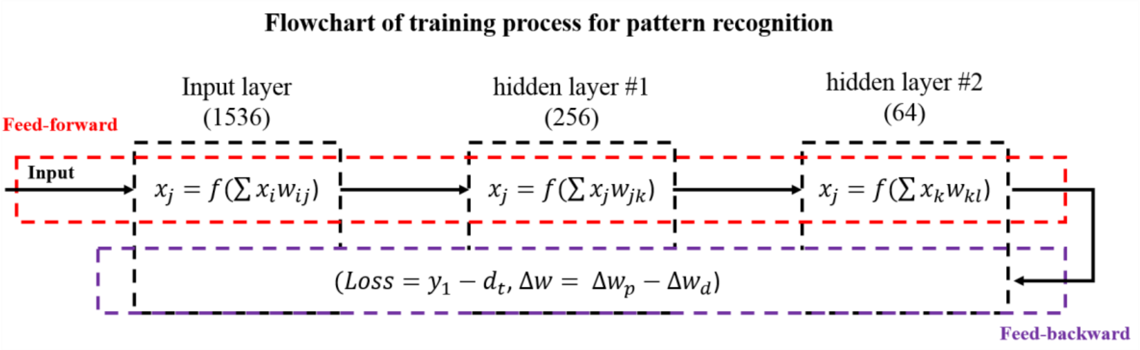

Figure S16. Flowchart of training process for pattern recognition in our multilayer artificial neural network.

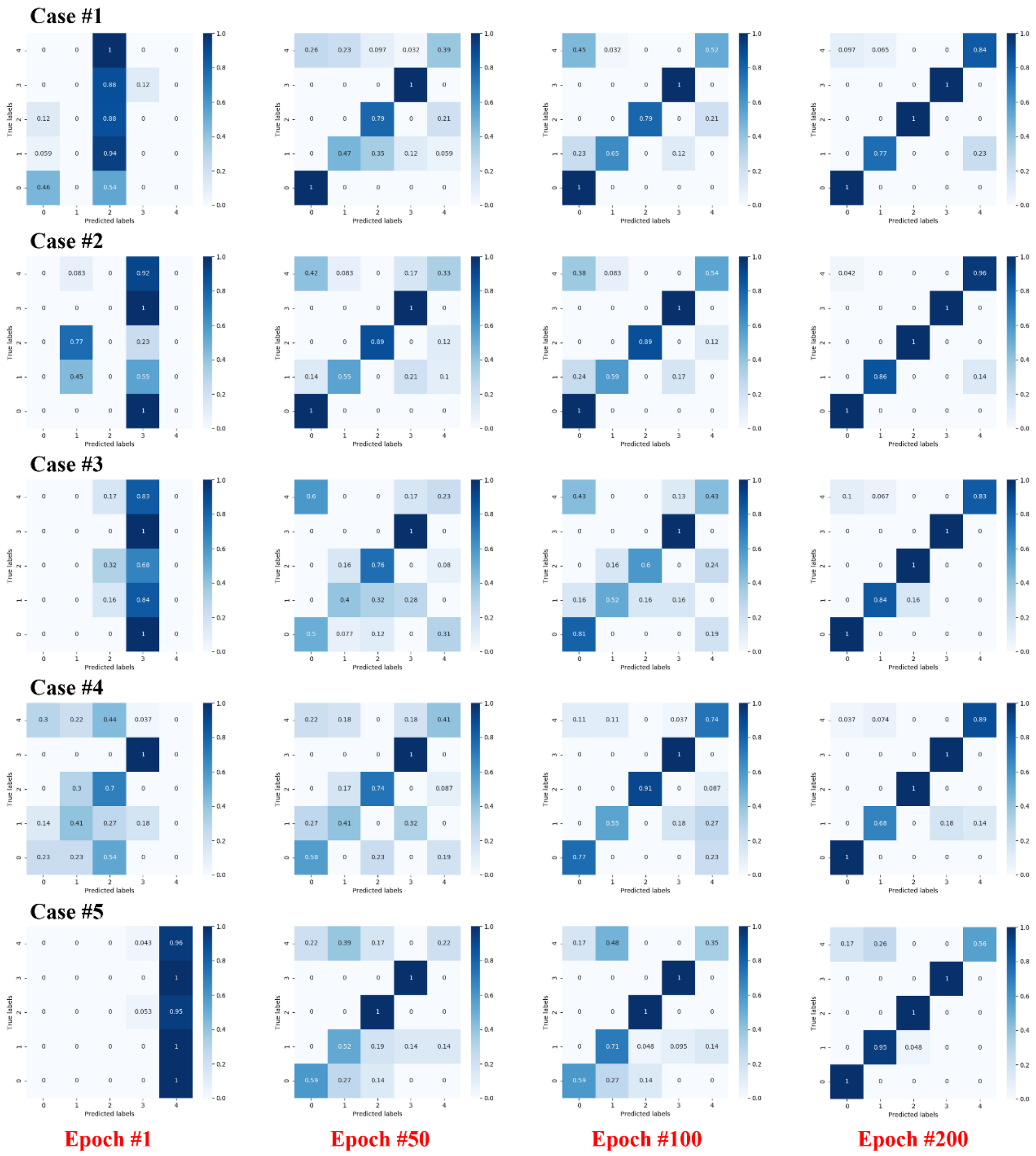

Figure S17. Confusion matrix for a classification test involving 125 PPG images with respect to the number of learning epochs, under different cases.

Supplementary Table

Table S1 Comparison of our work with previously-reported opto-electronic neuromorphic transistors in terms of the light bandwidth and plasticity adjusting model.

Structure	Light bandwidth	Plasticity adjusting model	Year	Ref.
P(VDFTrFE) /P(VP-EDMAEMAES) /PII-BT	Vis, NIR	Switchable plasticity[a]	2018	9
Si nanocrystals	NUV, Vis, NIR	Tunable plasticity[b]	2018	23
Pentacene /PMMA /$CsPbBr_3$	NUV, Vis	Switchable plasticity	2018	24
PEDOT: PSS/SnO_x/IGZO	NUV, Vis	Tunable plasticity	2019	25
Black phosphorus	NUV, Vis	Tunable plasticity[b]	2019	26
CuPc /p-6P	NUV, Vis	Switchable plasticity	2019	27
$MoSe_2$ /Bi_2Se_3	Vis, NIR	Tunable plasticity	2019	28
DPPDTT /$CsPbBr_3$ QDs	Vis	Tunable plasticity	2020	29
NT-CN /PMMA /pentacene	NUV, Vis	Switchable plasticity	2020	30
IGZO /ITO /$CsPbBr_3$ /PMMA	Vis	Tunable plasticity	2020	31
InGaCdO (IGCO)	NUV, Vis, NIR	Tunable plasticity	2020	32
MoS_2	NUV, Vis	Tunable plasticity	2020	33
C8-BTBT /F_{16}CuPc	NUV, Vis, NIR	Switchable plasticity		This work

[a] The plasticity switched by changing light wavelength.

[b] The plasticity tuned by changing light intensity, duration and times.

Table S2 Comparison of our work with previously-reported opto-electronic neuromorphic transistors in terms of the Morse code conversion.

Structure	Trigger way	Δt^{c}	Fluctuation[d]	Year	Ref.
FT4-DPP-based polymer NW	940 nm IR<br>360 nm UV	0.12 s	> 30%	2018	36
ZnO	Pressures (100 kPa)	0.009 s	> 50%	2020	3
Chitosan	Pressures (7.84 kPa)	0.5 s	> 200%	2020	37
C8-BTBT /PS /CPB QDs	365 nm UV	5 s	> 30%	2021	38
C8-BTBT /F_{16}CuPc	790-nm NIR	0.1 s	< 2%		This work

[c] The minimum interval between two triggers.

[d] The fluctuation of the response current triggered by the same command at Δt.

References

[3] H. Tan, Q. Tao, I. Pande, S. Majumdar, F. Liu, Y. Zhou, P. O. Å. Persson, J. Rosen, S. V. Dijken, *Nat. Commun.* **2020**, *11*, 1369.

[9] H. Wang, Q. Zhao Z. Ni, Q. Li, H. Liu, Y. Yang, L. Wang, Y. Ran, Y. Guo, W. Hu, Y. Liu, *Adv. Mater.* **2018**, *30*, 1803961.

[23] H. Tan, Z. Ni, W. Peng, S. Du, X. Liu, S. Zhao, W. Li, Z. Ye, M. Xu, Y. Xu, X. Pi, D. Yang, *Nano Energy* **2018**, *52*, 422-430.

[24] Y. Wang, Z. Lv, J. Chen, Z. Wang, Ye, Zhou, *Adv. Mater.* **2018**, *30*, 1870287.

[25] J. Yu, L. Liang, L. Hu, H. Duan, W. Wu, H. Zhang, J. Gao, F. Zhuge, T. Chang, H. Cao, *Nano Energy* **2019**, *62*, 772-780.

[26] T. Ahmed, S. Kuriakose, E. Mayes, R. Ramanathan, V. Bansal, M. Bhaskaran, S. Sriram, S. Walia, *Small* **2019**, *15*, e1900966.

[27] J. Zhu, Y. Yang, R. Jia, Z. Liang, W. Zhu, Z. U. Rehman, L. Bao, X. Zhang, Y. Cai, L. Song, R. Huang, *Adv Mater.* **2018**, *30*, 1800195.

[28] Y. Wang, J. Yang, W. Ye, D. She, J. Chen, Z. Lv, V. Roy, H. Li, K. Zhou, Q. Yang, *Adv. Electron. Mater.* **2019**, *6*, 1900765.

[29] D. Hao, J. Zhang, S. Dai, J. Zhang, J. Huang, *ACS Appl. Mater. Interfaces* **2020,** *12*, 39487.

[30] H.-L. Park, H. Kim, D. Lim, H. Zhou, Y.-H. Kim, Y. Lee, S. Park, T.-W. Lee, *Adv. Mater.* **2020**, *32*, e1906899.

[31] S. S. Periyal, M. Jagadeeswararao, S. E. Ng, R. A. John, N. Mathews, *Adv. Mater. Technol.* **2020**, *5*, 2000514.

[32] H. Duan, K. Javaid, L. Liang, L. Huang, J. Yu, H. Zhang, J. Gao, F. Zhuge, T.-C. Chang, H. Cao, *Phys. Status Solidi RRL* **2020,** *14*, 1900630.

[33] T. Wang, J. Meng, Z. He, L. Chen, H. Zhu, Q. Sun, S. Ding, P. Zhou, D. W. Zhang, *Adv. Sci.* **2020**, *7*, 1903480.

[36] Y. Lee, J. Y. Oh, W. Xu, O. Kim, T. R. Kim, J. Kang, Y. Kim, D. Son, J. B.-H. Tok, M. J. Park, Z. Bao, T. -W. Lee, *Sci. Adv.* **2018**, *4*, eaat7387.

[37] F. Yu, J. C. Cai, L. Q. Zhu, M. Sheikhi, Y. H. Zeng, W. Guo, Z. Y. Ren, H. Xiao, J. C. Ye, C. -H. Lin, A. B. Wong, T. Wu, *ACS Appl. Mater. Interfaces* **2020**, *12*, 26258-26266.

[38] Q. Shi, D. Liu, D. Hao, J. Zhang, L. Tian, L. Xiong, J. Huang, *Nano Energy* **2021**, *87*, 106197.
